# Derivative and Q-analysis Spectrophotometric Methods for Estimation of Hydrochlorothiazide and Olmesartan Medoxomil in Tablets

**DOI:** 10.4103/0250-474X.58176

**Published:** 2009

**Authors:** K. P. Bhusari, P. B. Khedekar, Seema Dhole, V. S. Banode

**Affiliations:** Department of Pharmaceutical Chemistry, Sharad Pawar College of Pharmacy, Wanadongri, Hingna Road, Nagpur-441 110, India; 1 Department of Chemical and Process Engineering, University of Sheffield, Sheffield S1 3JD, United Kingdom

**Keywords:** Derivative and Q-analysis spectrophotometric methods, hydrochlorothiazide, olmesartan medoxomil

## Abstract

Two methods for simultaneous estimation of hydrochlorothiazide and olmesartan medoxomil in combined tablet dosage form have been developed. The first method is the application of Q–analysis method (absorbance ratio), which involves the formation of Q–absorbance equation at 264 nm (isobestic point) and at 271 nm, the maximum absorption of hydrochlorothiazide. The linearity ranges for hydrochlorothiazide and olmesartan medoxomil were 2.5-22.5 μg/ml and 4-36 μg/ml, respectively. The second method is based on the derivative spectrophotometric method at zero crossing wavelengths. The linearity ranges for hydrochlorothiazide and olmesartan medoxomil were 2.5-20 μg/ml and 4-32 μg/ml, respectively. The accuracy of the methods were assessed by recovery studies and was found to be 100.45% ±0.4215 and 100.24% ±0.3783 for absorbance ratio method and 99.39% ±0.221 and 99.72% ±0.11 for first derivative method, for hydrochlorothiazide and olmesartan medoxomil, respectively. These methods are simple, accurate and rapid, those require no preliminary separation and can therefore be used for routine analysis of both drugs in quality control laboratories.

Hydrochlorothiazide (HCTZ), chemically 6-chloro-3,4-dihydro-2*H*-1,2,4-benzothiadi- azine-7-sulphonamide-1,1-dioxide, is a diuretic and antihypertensive drug, which inhibits the reabsorption of sodium and calcium at the beginning of distal convoluted tubules. The chemical structure of HCTZ is shown in fig. 1. The typical dose of HCTZ is 12.5 mg per day[1–5]. Literature survey revealed that HPLC, HPTLC and spectroscopic methods have been reported for its determination in combination with other drugs[6–17]. Olmesartan medoxomil (OLME), chemically (5-methyl-2-oxo-1,3-dioxolen-4-yl)methyl-4-(1-hydroxyl-1-methylethyl)-2-propyl-1-[4-{2-(tetrazol-5-yl)-phenyl}-phenyl]methylimidaz-ole-5-carboxylate is a prodrug used as antihypertensive, which blocks the vasoconstrictor effect of angiotensin-II by selectively blocking the binding of angiotensin-II to the AT_1_ receptor in vascular smooth muscle[18–21]. Literature survey reveals that capillary zone electrophoresis method is reported for its estimation alone and HPTLC method has been reported for its estimation in combination with HCTZ[22,23]. The dose of OLME is 20 mg daily and its structure is shown in fig. 2. A combination of drugs, HCTZ (12.5 mg) and OLME (20 mg) in tablet formulation is available commercially (Olmezest-H 20, Sun Pharmaceutical Industries Ltd., Mumbai, India). However, no spectrophotometric method has yet been reported for simultaneous estimation of HCTZ and OLME. Hence, an attempt has been made to develop and validate, in accordance with ICH guidelines, a simple, precise, accurate and economical spectrophotometric method for quantitative analysis of HCTZ and OLME in combined tablets[24,25].

**Fig. 1:**
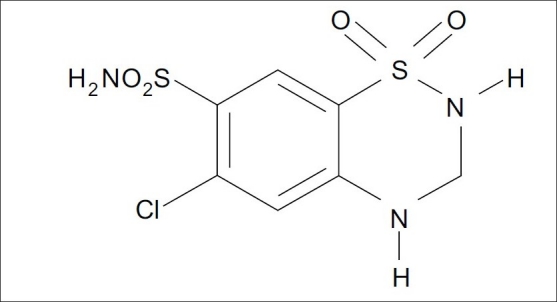
Hydrochlorothiazide

**Fig. 2:**
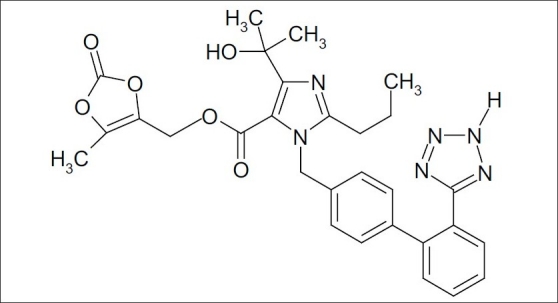
Olmesartan medoxomil

## MATERIALS AND METHODS

Pharmaceutically pure sample of HCTZ and OLME were obtained as generous gifts from Golden Cross Pvt. Ltd., Daman, India and MSN Laboratories Pvt. Ltd., Medak, India, respectively. Methanol AR grade (Merck Ltd., Mumbai, India) was used as solvent in the study. Double beam UV/Vis spectrophotometer, Shimadzu model 1601 with a pair of 10 mm matched quartz cells was used to measure absorbance of the resulting solution.

### Preparation of standard stock solution:

Accurately, 50 mg each of HCTZ and OLME was weighed separately and transferred to two different 50 ml volumetric flask. Each drug was dissolved in methanol and volume was made up to the mark with methanol. The standard stock solution (1000 μg/ml) were further diluted separately to obtain working standard solution of concentration 7.5 μg/ml of HCTZ and 12.0 μg/ml of OLME.

### Study of spectra and selection of wavelengths:

**Fig. 3:**
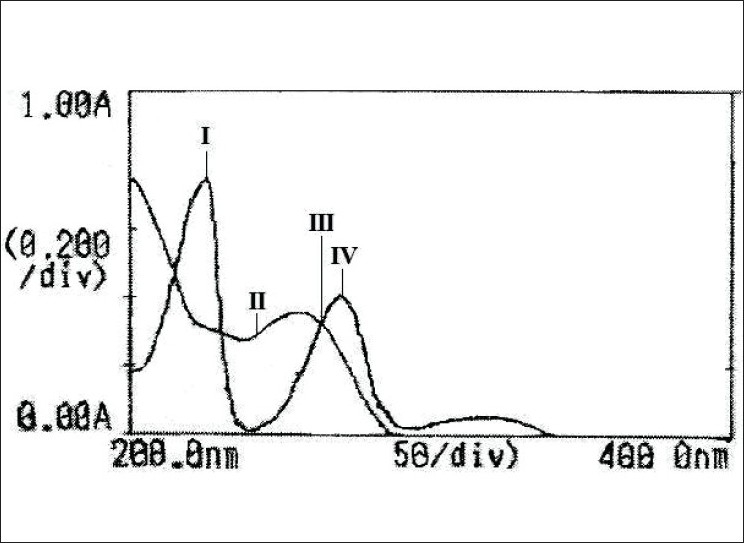
Overlain spectra of hydrochlorothiazide and olmesartan medoxomil I is hydrochlorothiazide; II is olmesartan medoxomil; III is isobestic point (264 nm) and IV is the λmax of hydrochlorothiazide (271 nm).

**Fig. 4:**
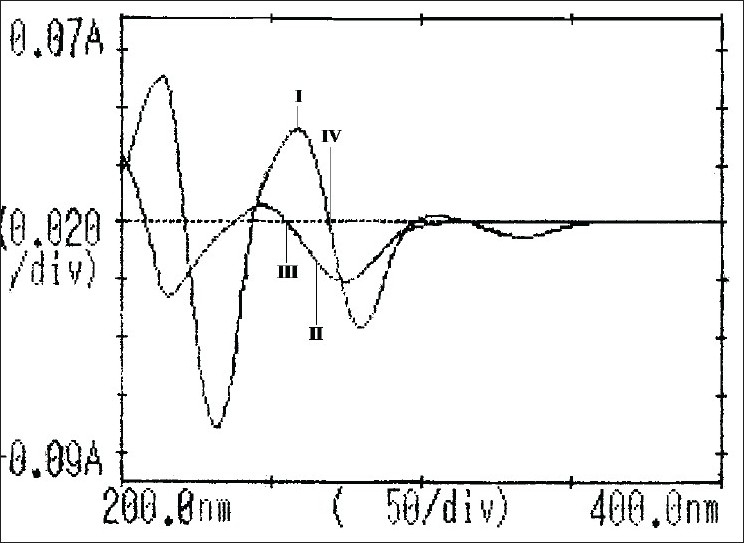
Overlain first order derivative spectra of hydrochlorothiazide and olmesartan medoxomil I is hydrochlorothiazide; II is olmesartan medoxomil; III and IV are zero crossing points of hydrochlorothiazide (269.5 nm) and olmesartan medoxomil (254.5 nm), respectively.

Each working standard solution was scanned between the range 200-400 nm in 1 cm cell against blank. The zero and first order derivative absorption spectra were recorded. Two wavelengths were selected from the overlain zero order spectra (fig. 3), 264 nm (Isobestic point) and 271 nm (λ_max_ of HCTZ) for formation of Q-absorbance equation. The peak amplitude of first derivative spectra (fig. 4) was measured at 254.5 nm and 269.5 nm for HCTZ and OLME, respectively.

### Procedure for analysis of tablet formulation:

Twenty tablets were accurately weighed and average weight was calculated. The tablets were triturated to a fine powder. An accurately weighed quantity of powder equivalent to 60 mg of OLME was dissolved in methanol and volume was made up to 50 ml. The solution was filtered through Whatmann filter paper No. 41 and aliquot portion of filtrate was diluted to produce solution of 7.5 μg/ml of HCTZ and 12 μg/ml of OLME. The absorbance of sample solution was measured at selected wavelengths and the concentrations of the two drugs were estimated using absorbance ratio and first order derivative methods. The analysis procedure was repeated six times and the results are depicted in Table 1.

**TABLE 1:** RESULTS OF ANALYSIS OF TABLET FORMULATION

Drugs	Q-analysis method % ±SD (n=6)	First order derivative method % ±SD (n=6)
HCTZ	100.23±0.360	99.86±0.428
OLME	100.34±0.416	99.90±0.486

SD is standard deviation. HCTZ is hydrochlorothiazide and OLME is olmesartan medoxomil.

## RESULTS AND DISCUSSION

In quantitative estimation of two components by Q-analysis method, absorbances were measured at the isobestic wavelength and maximum absorption wavelength of one of the two drugs. From overlain spectra of HCTZ and OLME (fig. 3), absorbances were measured at the selected wavelengths i.e., 264 nm (isobestic point) and at 271 nm, the maximum absorption of HCTZ. The absorptivity coefficients of each drug at both wavelengths were determined. The concentration of each drug in laboratory mixture and tablet formulation was determined by substituting the absorbance and absorptivity coefficients in the following sets of equations, Cx= Qm-Qy/Qx-Qy×A/Ax_1_ (Eqn. 1) and Cy= Qm-Qx/Qy-Qx×A/Ay_1_ (Eqn. 2), where, Cx is the concentration of HCTZ, Cy is the concentration of OLME, Qm is the ratio of absorbance of sample at selected wavelengths, Qx is the ratio of absorptivity coefficients of HCTZ, Qy is the ratio of absorptivity coefficients of OLME, Ax_1_ is absorptivity coefficient of HCTZ at 264 nm, Ay_1_ is absorptivity coefficient of OLME at 264 nm.

Upon examining the first derivative spectra of the two drugs (fig. 4), it can be noticed that HCTZ can be determined at 254.5 nm where OLME has no contribution and OLME can be determined at 269.5 nm where HCTZ shows a zero crossing. The concentrations of drugs were determined from the standard calibration curve of HCTZ and OLME, respectively by interpolation method. A= 0.0028c+0.0009, r= 0.9977 (λ= 254.5nm) (Eqn. 3) and A= 0.0016c+0.0002, r= 0.9998 (λ= 269.5nm) (Eqn. 4), where, c is the concentration in μg/ml, A is the peak amplitude of the first derivative curves at 254.5 nm and 269.5 nm for HCTZ and OLME respectively, r is correlation coefficient. The peak amplitude of first derivative spectra was measured at 254.5 nm and 26.5 nm for HCTZ and OLME, respectively. The amount of the two drugs was calculated from the computed regression Eqns. 3 and 4. The results are depicted in Table 1.

The methods were validated with respect to linearity, limit of detection (LOD), limit of quantification (LOQ), precision, accuracy and ruggedness. To study accuracy of the developed methods, recovery studies were carried out using standard addition method at three different levels. Percent recovery and low relative standard deviation value shows accuracy of the spectrophotometric methods. For precision of methods, the relative standard deviation for six replicates of sample solution was less than 2%, which met the acceptance criteria established for s pectrophotometric methods. Ruggedness of the proposed method was determined by analysis of sample solution prepared by proposed methods between different days and analysts. The percent relative standard deviation reported was found to be less than 2% showed ruggedness of the s pectrophotometric methods. The results obtained are summarized in Tables 2 and 3.

**TABLE 2:** RESULTS OF RECOVERY STUDIES IN TABLET FORMULATION

Method	Spike level (μg/ml)		Percent recovery % ± SD (n=6)	
		
	HCTZ	OLME	HCTZ	OLME
I	1.5	2.4	100.40±0.420	100.19±0.371
II	1.5	2.4	99.18±0.211	99.84±0.112
I	3.0	4.8	100.28±0.463	99.88±0.391
II	3.0	4.8	99.36±0.312	99.62±0.231
I	4.5	7.2	100.35±0.388	100.28±0.360
II	4.5	7.2	99.62±0.201	99.71±0.099

Method I is the Q-analysis method and Method II is the first order derivative. HCTZ is hydrochlorothiazide and OLME is olmesartan medoxomil.

**TABLE 3:** VALIDATION PARAMETERS

Parameters	Absorbance ratio method		First order derivative method	
		
	HCTZ	OLME	HCTZ	OLME
Slope	0.081	0.0325	0.0028	−0.0016
Intercept	0.0149	0.0038	0.00009	0.0002
Correlation coefficient	0.9997	0.9998	0.9997	0.9997
Linearity range	2.5-22.5 μg/ml	4-36 μg/ml	2.5-20 μg/ml	4-32 μg/ml
LOD(μg/ml)	0.58	0.61	0.6	0.7
LOQ(μg/ml)	0.95	1.2	0.9	1.1
Precision (% RSD)	0.359	0.414	0.416	0.483
Intra day (n=3) %± SD	100.33±0.088	100.63±0.121	99.70±0.711	99.62±0.705
Inter day (n=3) %± SD	99.78±0.224	99.23±0.297	100.22±0.890	100.23±0.761
Different analyst (n=3) %± SD	100.83±0.030	100.26±0.064	99.64±0.442	99.61±0.457

LOD is limit of detection; LOQ is limit of quantification; % RSD is percentage relative standard deviation. HCTZ is hydrochlorothiazide and OLME is olmesartan medoxomil.

The proposed UV spectrophotometric methods are a simple, accurate, precise, rapid and economical for the simultaneous estimation of HCTZ and OLME in tablet dosage form. The proposed methods use inexpensive reagents, solvents and instruments that are available in laboratories. Hence, these methods can be conveniently adopted for the routine analysis in quality control laboratories.
